# Selective Fair Behavior as a Function of Psychopathic Traits in a Subclinical Population

**DOI:** 10.3389/fpsyg.2017.01604

**Published:** 2017-09-13

**Authors:** Takahiro Osumi, Hideki Ohira

**Affiliations:** ^1^Department of Psychology, Hiroshima Shudo University Hiroshima, Japan; ^2^Department of Psychology, Nagoya University Nagoya, Japan

**Keywords:** psychopathic traits, fairness, reciprocity, punishment, social distance, dictator game, ultimatum game

## Abstract

Psychopathy is a group of personality traits that are associated with violations of social norms. Previous studies have suggested that people with psychopathic traits in subclinical populations do not necessarily display antisocial, self-defeating behaviors, and instead may strategically show adaptive behaviors in response to cues during reciprocal social interactions. Therefore, in the present study, we examined whether the association between psychopathic traits and unfair behavior can be moderated by a potential for punishment and social distance (anonymity), which are known to facilitate fair behavior. We focused on two psychopathic traits: primary and secondary psychopathy. Primary psychopathy is characterized by callousness, shallow affect, manipulation, and superficial charm. In contrast, secondary psychopathy is associated with impulsivity and lack of long-term goals, and is related to hostile behavior. A total of 348 undergraduate students determined the amounts of money that they would offer to strangers or friends at their university in hypothetical scenarios of the ultimatum game (UG) and the dictator game (DG). While gender affected decisions in the hypothetical scenarios of the DG, it did not interact with psychopathic traits. The score for primary psychopathy on the Levenson self-report psychopathy scale predicted unfair monetary offers to strangers in the DG, where participants could not be punished. However, compared with their offers in the DG, individuals with higher scores for primary psychopathy made larger offers in the UG, where low offers could trigger punishment from the recipient. Moreover, primary psychopathy did not decrease the amounts of offers in either game when the participant considered the recipient to be a friend. On the other hand, secondary psychopathy was not associated with differences in behavioral fairness depending on a potential for punishment or social distance. Based on these findings, we discuss strategic social skills as a function of primary psychopathy.

## Introduction

In social life, a serious problem of psychopathy is the violation of social norms. For example, individuals with psychopathy tend to commit and repeat criminal behaviors more frequently than non-psychopathic individuals (Hare et al., 2000). However, psychopathy is not limited to criminal or clinical contexts (Cleckley, 1988), and psychopathic traits are believed to be continuously distributed in the general population (Edens et al., 2006). These clinical and empirical findings imply that individuals in the general population who exhibit high levels of psychopathic traits might exhibit immoral or antisocial acts without being noticed since they may disguise parts of their personalities, such as egocentricity, callousness, and irresponsibility, and superficially comply with social norms or behave in socially acceptable ways. Particularly, as can be seen in an item on a self-report scale for assessing psychopathic traits, i.e., “I tell other people what they want to hear so that they will do what I want them to do” (Levenson et al., 1995), psychopathic traits can be associated with superficial displays of socially desirable actions, but only against a background of self-interest. Therefore, studies on conditions that moderate the association between psychopathic traits and the violation of social norms should contribute to an understanding of their adaptive and maladaptive functions.

Fairness is a social norm that underlies reciprocal interactions within social groups. For example, individuals who engage in unfair behavior can be punished by other individuals. Under this threat of negative reciprocity, fair behavior helps individuals avoid punishment in interpersonal interactions. The ability of a potential for punishment to increase behavioral fairness can be illustrated by comparing behavioral performance in the dictator game (DG) to that in the ultimatum game (UG). In the DG, in which a sum of money is divided between two players, one player unilaterally decides the amounts of money to be distributed to him/herself and the second player. Both players are then assigned money based solely on the first player’s decision. Conversely, in the UG, the second player (as the responder) can decide whether to accept or reject the monetary offer made by the first player. If the responder accepts the offer, then the deal goes forward. However, if the responder rejects the offer, then neither player receives any money. Research has shown that approximately half of responders reject unfair offers that are less than 20% of the total pool of money (Camerer, 2003). Such rejection is generally considered to be punishment or revenge for norm violators (e.g., Strobel et al., 2011), and it has been shown that this threat of punishment generally increases the amount of the first player’s offer in the UG. Empirical data indicated that the first player in the DG distributes approximately 20% of a stake to the second player (Forsythe et al., 1994; Camerer, 2003), while approximately 40% of the stake is offered in the UG (Güth et al., 1982; Camerer, 2003).

A further understanding of how behavior can be used to avoid negative reciprocity can be obtained by focusing on behavioral fairness in an interaction with someone who is socially close. Fairness serves as a criterion upon which to evaluate whether a person is trustworthy, which is an essential psychological facilitator of mutual cooperation and reciprocal altruism over the course of multiple interactions (e.g., Berg et al., 1995). According to this view, in addition to the punishment or revenge that is possible in “one-shot” social interactions, violations of fairness norms can also have a harmful effect on future interactions with the same person. Therefore, behavioral fairness is likely to be modulated depending on the nature of the relationship with others. For instance, in the DG, the first player’s offer is modulated by the social distance to the recipient, in that a close recipient is treated more fairly than a distant, anonymous recipient (Hoffman et al., 1996; Rankin, 2006; Charness and Gneezy, 2008). Also, the psychological responses of the recipients of unfair offers are influenced by the social distance to the proposers, in that unfair offers from a close person are more likely to cause dissatisfaction compared to those from strangers (Wu et al., 2011). These findings suggest that a person’s consideration of fairness is increased in interactions with others who are socially closer to them.

It is unclear whether individuals with high tendencies toward psychopathic traits focus on an instant economic reward despite the situation, or whether they take into account personal relations to avoid negative reciprocity. They will be likely to display unfair behaviors even toward a close person with whom they should have additional interactions in the future if they can not predict and respond to adverse consequences of unfair behaviors. Based on a finding of reduced physiological reactivity associated with aversive events (Lykken, 1957; Patrick et al., 1993; Levenston et al., 2000), it has been argued that a low level of fearfulness (Lykken, 1995) or deficient functioning in the defensive system (Patrick, 2007), which reduces emotional responses to potential punishment, underlies aggressiveness with norm violations in individuals with psychopathy. In addition, multiple studies have suggested that defensive dysfunction is specifically associated with primary (low-anxiety) psychopathy or interpersonal/affective features, including callousness, cunning, and a lack of empathy and/or remorse, rather than secondary (neurotic) psychopathy or aspects of deviant behaviors such as impulsivity and poor behavioral control in both criminal and community samples (e.g., Patrick, 1994; Benning et al., 2005). Accordingly, high tendencies toward primary psychopathy may prompt unfair behaviors despite a potential for punishment in interpersonal interactions.

This possibility has not been supported by previous findings regarding the first player’s behavior in the DG and the UG, though those studies have been limited with respect to the sample size or male-female ratio. Koenigs et al. (2010) found that offenders who scored high in primary psychopathy (*n* = 6) offered smaller amounts of money to their partners in the DG compared to both non-psychopathic offenders (*n* = 22) and offenders with secondary psychopathy (*n* = 6). However, they made relatively fair offers, similar to those of other offenders, in the UG. In a study by Gillespie et al. (2013), which recruited 60 participants (10 males and 50 females) from a university, primary psychopathy did not influence monetary offers either the DG or UG. These findings suggest that, at the behavioral level at least, individuals with high tendencies toward primary psychopathy show reactivity to a potential for punishment. If this is the case, we can predict that they will be less likely to propose unfair offers in both the DG and UG when their partner is a friend compared to when their partner is a stranger.

Furthermore, behavioral fairness modulated by social distance might be associated with secondary psychopathy. In contrast to the unemotional features of primary psychopathy, subjects with secondary psychopathy are characterized as being neurotic and exhibiting anxiety (Karpman, 1941; Skeem et al., 2003). In addition, secondary psychopathy is associated with anger and hostile behaviors in response to adverse events in interpersonal interactions (Hicks and Patrick, 2006). Mealey (1995) proposed that the aggressiveness seen in secondary psychopathy (sociopathy) originates from bad interpersonal experiences in early developmental stages, such as abusive treatment from others. Therefore, individuals who score high in secondary psychopathy may exhibit a behavioral strategy in which they respond at a competitive disadvantage. Based on this hypothesis, we can predict that secondary psychopathy leads a person to display unfair, aggressive behaviors especially toward unfamiliar people or those who are in an adversarial position. On the other hand, in interactions with reliable and cooperative people, secondary psychopathy may not increase harsh behaviors. In support of these predictions, Gillespie et al. (2013) revealed that a group bias was exaggerated as a function of secondary psychopathy, in that in-group members (students from the same university) were treated more fairly than out-group members (students from other universities) in both DG and UG. However, it has been unclear whether psychopathic traits modulate behavioral fairness in response to social distance (e.g., anonymity) in interpersonal interactions within a group.

The present study examined how primary and secondary psychopathy affect behavioral fairness in response to not only a potential for punishment in a one-shot interaction, but also the social distance to the partner in the interaction. Therefore, we used the DG and the UG, where the recipient was either a stranger or a friend. The potential for punishment in a one-shot interaction was manipulated by whether the participants performed the DG (absence) or the UG (existence), while social distance was manipulated by whether the participants interacted with a stranger (one-shot relationship) or a friend (continuing relationship). Moreover, this study aimed to collect data from a larger sample relative to previous studies to improve the power of the statistical tests. Therefore, the study was simplified by the use of hypothetical scenarios. This approach might reduce the impact of the experimental setting on exchanges of convenience, and some studies have used hypothetical scenarios to provide meaningful findings regarding moral reasoning (e.g., Koenigs et al., 2012).

Two primary hypotheses were evaluated. First, individuals with primary psychopathy may be sensitive to the adverse consequences of unfair behaviors for economic self-interest and personal relationships. If this is the case, primary psychopathy should be associated with unfairly small monetary offers in the DG, but not in the UG when the partner is hypothesized to be a stranger. In addition, primary psychopathy might not be associated with unfair offers in either game when the hypothesized partner is a friend. Second, secondary psychopathy may increase hostile behaviors especially to unfamiliar people. Therefore, consistent with the report by Gillespie et al. (2013), secondary psychopathy was predicted to be associated with unfair monetary offers in both the DG and the UG with a stranger, but not in games with a friend. Moreover, we examined whether or not the effects of psychopathic traits on hypothetical social interactions are gender-specific.

## Materials and Methods

### Participants

A total of 349 Japanese undergraduate students voluntarily completed questionnaires during part of a class session at the university. Data regarding 348 participants (228 males) between 18 and 29 years of age (*M* = 18.63; *SD* = 1.28) were then analyzed after one participant was excluded due to missing values.

### Psychopathic Traits

The Japanese version of the Levenson Self-Report Psychopathy Scale (LSRP; Levenson et al., 1995) was used to assess psychopathic traits. The LSRP is a self-report questionnaire that uses a four-point Likert scale to assess psychopathic traits in non-institutionalized populations (“1: disagree strongly”; “2: disagree somewhat”; “3: agree somewhat”; “4: agree strongly”). The items are divided into two factors: primary and secondary psychopathy. The primary psychopathy subscale, which consists of 16 items, reflects interpersonal and affective features including manipulation, egocentricity, and a lack of empathy and/or remorse. In contrast, the secondary psychopathy subscale, which consists of 10 items, addresses social deviance behaviors such as impulsivity, stimulation seeking, and poor behavioral control.

The Japanese version of the LSRP was developed using back translation for each item (Sugiura and Sato, 2005). This version includes the same factor structure as the original, and has been shown to possess construct validity and adequate test–retest reliability (Osumi et al., 2007a). The coefficient alphas were 0.75 for primary psychopathy and 0.58 for secondary psychopathy, which are approximately equivalent to those in past studies. In the report by Levenson et al. (1995), the alphas for the primary psychopathy and secondary psychopathy subscales were 0.82 and 0.63, respectively. For Japanese samples, the alphas were 0.78 ∼0.80 for primary psychopathy and 0.56 ∼0.61 for secondary psychopathy (Osumi et al., 2007b, 2012; Osumi and Ohira, 2010; Masui et al., 2011). Overall, the alphas for secondary psychopathy on the LSRP in the previous and present studies are consistently low. However, Levenson et al. (1995) reported that the alpha for secondary psychopathy is probably acceptable for a 10-item scale. For the current sample, Pearson’s correlation coefficient between primary and secondary psychopathy was 0.30 (*p* < 0.001).

### Task and Procedure

Four hypothetical scenarios involving economic decision-making (see Supplementary Material) were presented to each participant. Therefore, participants were informed that they would not really be paid according to their decisions. At the beginning of each scenario, the participants were asked to imagine a situation in which they would divide an amount of money (Japanese 1,000 yen) between themselves and another person (the partner). In addition, participants were asked to imagine their partner in the game. In the stranger condition, they were instructed that the partner was an unknown student from the same university with whom the participant would not interact again, whereas in the familiar condition, the partner was a friend at the university. If participants were instructed that they should imagine a particular friend, the properties of different friends (e.g., attractiveness and social distance from the friend) might differ. Thus, to control for such individual differences, participants had to imagine a stranger and a friend in the abstract. Therefore, we did not specifically instruct participants to imagine a particular person in either condition. Moreover, the participants read a description of the scenario that differed depending on the type of game. For the DG, the participants were informed that they would decide how to divide an amount of money to be received by themselves and their partner, who would be unable to change the amount offered. Conversely, for the UG, the participants and their partners would receive money if the partner accepted how the money was to be divided. Otherwise, neither party would receive any money. Accordingly, there were four types of scenarios: (1) the DG with a stranger; (2) the DG with a friend person; (3) the UG with a stranger; and (4) the UG with a friend.

The participants made only one offer in a scenario per page. The participants began reading the scenarios together and then had to make a decision for each scenario within 20 s. They were free to choose any integral to offer up to 1,000 yen (e.g., 501 yen, 999 yen, or 0 yen, but not 543.21 yen). After 20 seconds for a scenario, the participants turned a page following the instructions of the experimenter, and began reading another scenario. The order of the four scenarios was counterbalanced between the participants.

After these decision tasks, participants completed the LSRP. The effect of order on measurements (decision-making tasks and the LSRP) was worth considering. To our knowledge, no previous study has reported that decision-making tasks that include social interactions can affect the scores of primary and secondary psychopathy, at least as measured by the LSRP. We considered the possibility that personality questionnaires may make participants more aware of the association between personality and behavioral performance in the tasks, and thus modify their behavior to be consistent with their responses to the question items.

## Results

### Gender Differences

**Table 1** shows the scores of primary and secondary psychopathy and the amounts of money offered in each condition for males and females. First, we examined gender differences in the two psychopathic traits. According to analyses of variance, compared with female participants, male participants showed higher scores for both primary psychopathy [*F*(1,346) = 31.67, *p* < 0.001, $\eta_{p}^{2}$= 0.084) and secondary psychopathy [*F*(1,346) = 3.92, *p* < 0.05, $\eta_{p}^{2}$ = 0.011].

**Table 1:** Characteristics of male and female participants.

	Male	Female	Total
*N*	228	120	348
Age	18.65 (1.13)	18.60 (1.53)	18.63 (1.28)
**LSRP scores**			
Primary psychopathy	35.08 (5.70)	31.50 (5.54)	33.85 (5.98)
Secondary psychopathy	21.31 (3.52)	20.50 (3.82)	21.03 (3.65)
**Monetary offers**			
DG with stranger	344.56 (220.91)	442.92 (165.76)	371.51 (206.73)
DG with friend	445.39 (144.00)	492.50 (53.71)	461.63 (122.71)
UG with stranger	489.06 (115.11)	501.67 (78.57)	493.41 (104.02)
UG with friend	498.86 (80.39)	501.67 (26.67)	499.83 (66.88)

*DG, dictator game; UG, ultimatum game. Standard deviations are in parentheses. Scores of primary and secondary psychopathy traits are reported in points and monetary offers are in yen, out of a total amount of 1000 yen.*

Moreover, to examine gender differences in the amounts of offer, an analysis of variance was performed with game (DG or UG) and recipient (stranger or friend) as within-participant factors and gender as a between-participants factor. The results showed a significant main effect of gender, indicating that males offered smaller amounts of money to recipients than females [*F*(1,346) = 12.82, *p* < 0.001, $\eta_{p}^{2}$ = 0.036]. In addition, based on a significant interaction between game and gender [*F*(1,346) = 12.97, *p* < 0.001, $\eta_{p}^{2}$ = 0.036], the difference in offer between males and females was found in the DG [*F*(1,346) = 15.99, *p* < 0.001, $\eta_{p}^{2}$ = 0.044], but not in the UG [*F*(1,346) = 0.92, *p* = 0.37, $\eta_{p}^{2}$ = 0.003]. The partner × gender interaction and game × partner × gender interaction were not significant [*Fs*(1,346) < 2.52, *ps* > 0.11].

### Testing Hypotheses

To examine whether the effects of potential for punishment and social distance on monetary offers were modulated by psychopathic traits, we used hierarchical linear modeling (HLM; Raudenbush and Bryk, 2002). In HLM, the first-level variables are nested within the second-level variables. In addition, the first-level variables can be at the within-individual level of analysis, whereas the second-level variables can be at the between-individual level of analysis. Our data set included 348 participants, each of whom made monetary offers in four different scenarios. Thus, the Level 1 variables included repeated measures of game and partner and their interaction. The Level 2 variables included individual differences in the scores of primary and secondary psychopathy, gender and their interactions. Random effects were hypothesized for all Level 1 variables. Next, cross-level interactions were assessed by estimating fixed effects with robust standard errors. We used HLM 7.01 software (Raudenbush et al., 2013) to analyze the multilevel data. For each categorical variable, effects coding was applied (game: DG = -1, UG = 1; partner: stranger = -1, friend = 1; gender: male = -1, female = 1). Moreover, coding for gender was weighted based on the male-female ratio in the participants. Primary psychopathy, secondary psychopathy and all interaction terms were centered at the grand-mean.

Before testing our hypotheses with HLM, we assessed systematic within- and between-individual variance for the dependent variable (offer size) by estimating a null model. If there is no between-individual variance in the dependent variable, then HLM is not appropriate for data analyses because within-individual variance is enough to predict the dependent variable. As shown in **Table 2**, the null model results indicated that there was significant between-individual variance in the dependent variable and that an intra-class correlation coefficient (τ_00_/[τ_00_ + σ^2^]) was 0.168. That is, 16.8% of the variance in offers was between-individual. These results suggest that HLM was appropriate for analyzing these data and that there was between-participant variability in the amounts of money offered to partners in the scenarios.

**Table 2:** Parameter estimates and variance components of null model for monetary offers.

	Intercept (γ_00_)	Within-individual variance (σ^2^)	Between-individual variance (τ_00_)	Intra-classcorrelation coefficients
Dependent variables (offer size)	456.62^∗∗∗^	17327.73	3510.16^∗∗∗^	0.168

*^∗∗∗^*p* < 0.001.*

**Table 3** highlights the results of HLM predicting the amount of money offered in hypothetical scenarios. The aim of the present study was to assess cross-level moderating effects indicating that the Level 2 individual difference in psychopathic traits moderated the Level 1 effects of game and partner on the monetary amount of offers. We did not find any cross-level interactions with secondary psychopathy. In contrast, primary psychopathy had significant interactions with game (γ = 1.80, *SE* = 0.67, *t* = 2.70, *p* < 0.01) and partner (γ = 2.30, *SE* = 0.48, *t* = 4.79, *p* < 0.001). Moreover, there was a significant two-way interaction between primary psychopathy, game and partner (γ = -1.21, *SE* = 0.47, *t* = -2.57, *p* < 0.05).

**Table 3:** Fixed effects of cross-level interactions.

Level 1	Level 2	Coefficient	*SE*	*t*-Value	*p*-Value
Game	PP	1.80	0.67	2.70**	0.007
	SP	–0.30	1.28	–0.24	0.814
	Gender	–10.57	3.79	–2.79**	0.006
	PP × SP	–0.04	0.19	–0.19	0.847
	PP × Gender	0.47	0.66	0.72	0.475
	SP × Gender	–1.29	1.24	–1.04	0.300
	PP × SP × Gender	0.03	0.17	0.16	0.877
Partner	PP	2.30	0.48	4.79***	<0.001
	SP	–0.94	0.86	–1.09	0.279
	Gender	–1.72	3.19	–0.54	0.589
	PP × SP	0.19	0.13	1.44	0.152
	PP × Gender	0.32	0.49	0.65	0.516
	SP × Gender	–0.49	0.86	–0.57	0.569
	PP × SP × Gender	0.02	0.13	0.19	0.852
Game × Partner	PP	–1.21	0.47	–2.57*	0.011
	SP	0.35	0.79	0.45	0.655
	Gender	0.32	2.85	0.11	0.910
	PP × SP	–0.11	0.13	–0.87	0.386
	PP × Gender	–0.31	0.51	–0.60	0.546
	SP × Gender	0.05	0.86	0.06	0.954
	PP × SP × Gender	0.03	0.13	0.22	0.829

*PP, primary psychopathy; SP, secondary psychopathy. Degree of freedom for each effect was 340. ^∗∗∗^*p* < 0.001; ^∗∗^*p* < 0.01; ^∗^*p* < 0.05.*

**FIGURE 1:**
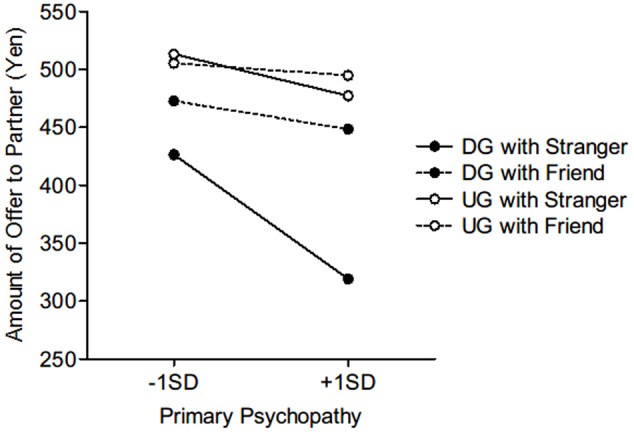
Effect of primary psychopathy on monetary offers (for a total amount of 1000 yen) in the dictator game (DG) and the ultimatum game (UG) with strangers and friends.

As illustrated in **Figure 1**, when the partner was a stranger, there was a simple interaction between game and primary psychopathy (γ = 3.02, *SE* = 1.01, *t* = 2.99, *p* < 0.01). Follow-up simple slope tests indicated that primary psychopathy predicted reduced offers in both DG (γ = -9.11, *SE* = 1.87, *t* = -4.86, *p* < 0.001) and UG (γ = -3.07, *SE* = 0.99, *t* = -3.09, *p* < 0.01). On the other hand, when the recipient was a friend, the simple interaction between game and primary psychopathy was not significant (γ = 0.59, *SE* = 0.56, *t* = 1.05, *p* = 0.29). In fact, while primary psychopathy had a marginally significant effect to reduce the amount of the offer in the DG with a friend (γ = -2.08, *SE* = 1.22, *t* = -1.70, *p* = 0.09), primary psychopathy did not significantly affect the offers in the UG with a friend (γ = -0.90, *SE* = 12, *t* = -1.10, *p* = 0.27).

Furthermore, we found a simple interaction between game and partner for individuals who scored high on primary psychopathy (γ = -27.88, *SE* = 4.33, *t* = -6.44, *p* < 0.001). *Post hoc* tests revealed that they offered a significantly larger amount of money in the UG than in the DG regardless of their partner (stranger: γ = 79.00, *SE* = 8.90, *t* = 8.88, *p* < 0.001; friend: γ = 23.22, *SE* = 5.14, *t* = 4.52, *p* < 0.001). In addition, the amount of money that they offered to a friend was significantly greater than that they offered to a stranger, in both games (DG: γ = 64.59, *SE* = 8.16, *t* = 7.92, *p* < 0.001; UG: γ = 8.82, *SE* = 4.02, *t* = 2.19, *p* < 0.05).

For individuals with low tendencies in primary psychopathy, again, there was a simple interaction between game and partner (γ = -13.60, *SE* = 3.41, *t* = -3.99, *p* < 0.001). Regardless of the partner, the amount of their UG offer was significantly larger than that of their DG offer (stranger: γ = 43.47, *SE* = 7.63, *t* = 5.70, *p* < 0.001; friend: γ = 16.26, *SE* = 4.79, *t* = 3.40, *p* < 0.001). Moreover, the size of the offer was increased when the recipient was a friend compared to when the recipient was a stranger in the DG (γ = 23.24, *SE* = 6.05, *t* = 3.84, *p* < 0.001), but not in the UG (γ = -3.97, *SE* = 3.36, *t* = -1.18, *p* = 0.24).

Gender had a significant interaction with game (γ = -10.57, *SE* = 3.79, *t* = -2.79, *p* < 0.01). Specifically, males made smaller offers than females in the DG (γ = 23.30, *SE* = 7.53, *t* = 3.10, *p* < 0.01), but not in the UG (γ = 2.14, *SE* = 3.59, *t* = 0.60, *p* = 0.55). However, as shown in **Table 3**, interactions between gender and psychopathic traits were not significant. In other words, the effects of psychopathic traits on monetary offers for males were not significantly different from those for females.

## Discussion

In the present study, we assessed whether psychopathic traits reduced behavioral fairness in interpersonal interactions even if the participant imagined the possibility of negative feedback from a partner. Particularly, we tested the effects of a potential for punishment in a one-shot interaction and the social distance to the partner of the interaction. Several parts of the present findings failed to support our predictions about selective fairness as a function of psychopathic traits. First, inconsistent with previous studies (Koenigs et al., 2010; Gillespie et al., 2013), primary psychopathy was associated with lower monetary offers to a stranger, not only in the DG, where participants can not be punished by recipients, but also in the UG, where there is a possibility of being punished. These differences may be due to our use of a hypothetical scenario and/or a larger sample size. Moreover, primary psychopathy tended to be associated with smaller offers, specifically in the DG, even if participants imagined that the partner was their friend. These findings suggest that primary psychopathy is associated with general deficits in the sense of fairness. However, secondary psychopathy was not associated with any differences in behavioral fairness depending on the potential for punishment and relationships with others. Although our findings are based on decision-making in hypothetical scenarios, our results may provide insight into adaptive and maladaptive social functions of psychopathic traits.

The present findings did not exclude the possibility that individuals with high psychopathic traits increase behavioral fairness depending on conditions. This study is the first to show that social distance can moderate the association between primary psychopathy and unfairness in monetary offers. In particular, despite a reduced sense of fairness, individuals who scored high for primary psychopathy offered more money to their friend than to a stranger even if there was no possibility of punishment in a one-shot interaction. Thus, they gave a higher priority to their relationship with a familiar person than to immediate economic rationality.

Reciprocity is a key concept to account for the adaptive role of fair, altruistic behaviors in direct social interactions within long-standing relationships (Trivers, 1971). In theory, people can behave altruistically toward a trustworthy person who is likely to return the altruistic behavior. Meanwhile, in a situation known as negative reciprocity, people are likely to penalize someone who has shown selfish behavior since there is a high risk that the latter may take advantage of the former’s altruism in the future. Based on this perspective, our findings imply that individuals who score high for primary psychopathy seek to avoid later negative feedback from a familiar person with whom they will remain in contact after a one-shot interaction. This interpretation is incompatible with the finding that primary psychopathy is associated with an increase in non-cooperative choices in an iterated prisoner’s dilemma game with a partner (Rilling et al., 2007), but compatible with another finding that individuals who score higher for primary psychopathy in a subclinical population are more likely to selectively exhibit non-cooperative behavior in a one-shot prisoner’s dilemma game when there are no cues regarding the possibility of future interactions with the partner, but not in the presence of such cues (Gervais et al., 2013).

Moreover, interactions that are not under a condition of anonymity can indirectly impact trust relationships with third parties via reputation. Thus, it has also been suggested that, from the viewpoint of indirect reciprocity (Nowak and Sigmund, 1998), fair behavior in interactions with friends is important for adaptive social life within a group. Therefore, primary psychopathy, at least in the general population, appears to be associated with the ability to lead an adaptive social life through both direct and indirect interactions.

The association between primary psychopathy and lower offers in the UG might be the result of general deficits in a sense of fairness, rather than a reduced sensitivity to a potential for punishment because individuals who scored high for primary psychopathy increased the amounts they offered to a stranger in the UG relative to those in the DG. Thus, the present study suggests that individuals with a high tendency for primary psychopathy can moderate the unfairness of their interpersonal behaviors when there is a potential for punishment, which appears to be inconsistent with previous hypotheses regarding low levels of fearfulness (Lykken, 1995) and defensive dysfunction (Patrick, 2007) in primary psychopathy.

Newman’s response modulation theory (Patterson and Newman, 1993; Newman and Lorenz, 2003) may help to explain our findings. He and his colleagues found that the association between primary psychopathy and reduced sensitivity to punishment is moderated by the allocation of attentional resources to information relevant to punishment (Newman et al., 2010; Baskin-Sommers et al., 2011). Hence, because individuals who score higher for primary psychopathy are intrinsically insensitive to punishment, they need top–down control to focus on the adverse consequences of unfair behavior. Thus, they may be able to exhibit fair behavior if they realize the association between norm violations and punishment. In addition, the interpersonal/affective features of psychopathy, which correspond to primary psychopathy and are associated with increased executive function may be important here (Sellbom and Verona, 2007; Feilhauer et al., 2012). Accordingly, the present results suggest that, under cognitive control, individuals with a high tendency for primary psychopathy achieve their goals not through excessively unfair behaviors that are likely to be targeted for punishment, but rather through slightly unfair behaviors that are relatively acceptable.

Another possibility involves the selective empathic processing in psychopathic traits. According to Blair (1995), impaired processing of the distress of others leads to a failure by psychopathic individuals to inhibit violent behaviors. However, the selective fairness behaviors in response to a potential for punishment may be enabled by an understanding that other individuals will have negative feelings from receiving unfair treatment and some will probably seek revenge. In the UG, the rejection of an unfair offer is associated with anger (Pillutla and Murnighan, 1996). Furthermore, meta-analyses have reported that psychopathy is consistently associated with deficits in recognizing others’ expressions of fear and sadness, but not of anger or disgust (Marsh and Blair, 2008; Dawel et al., 2012). Thus, selective empathic processing of such emotions may help individuals who score high for primary psychopathy increase their attention to adverse consequences of unfair behaviors and enhance their decisions to engage in fair behavior to avoid punishment or revenge from others.

Our results were inconsistent with those in a previous study which reported that individuals who score high for secondary psychopathy are sensitive to personal relationships (Gillespie et al., 2013). This discrepancy may be due to the low reliability of the secondary psychopathy subscale of the LSRP. To investigate the effect of secondary psychopathy more accurately, future studies should need to improve reliability of measuring secondary psychopathy. Otherwise, because most of the participants in the study by Gillespie et al. (2013) were females, the effect of secondary psychopathy on the sensitivity to a personal relationship might be gender-specific. However, we did not find any interactions of gender with the effects of psychopathic traits. Furthermore, the current study was different from the previous study in that the relationship with the recipient varied in terms of the social distance with in-group members (strangers vs. friends), but not according to group membership (out-group vs. in-group members). Gillespie et al. (2013) reported that secondary psychopathy was associated with reduced behavioral fairness during interactions with out-group members relative to in-group members, which supports the notion that secondary psychopathy is associated with high levels of hostility (Hicks and Patrick, 2006). Apparently, strangers within the participant’s in-group did not arouse a feeling of hostility, and thus secondary psychopathy had no effect on behavioral fairness in interactions with these recipients.

The present study has several limitations to consider. A major limitation is that the current results are based on hypothetical scenarios. Therefore, it is still unclear whether real situations might induce greater self-interest that would drive norm violations while disregarding the potential for punishment or social relationships. Also, decisions in the hypothetical scenarios by no means cause participants to lose money or break personal relationships in real life, and thus the emotional responses that could be induced by the scenarios might be different from those in reality. Rather, the decisions in the scenarios might represent reference attitudes to a potential for receiving punishment from others or breaking a relationship with a close person. Moreover, the hypothetical scenarios by a within-participant design might have accidentally cued participants to be aware of the purpose of the study. The nature of our instructions might have biased the participants’ decisions to obscure the effect of psychopathic traits. In particular, participants might think that they should decide fairly in response to a potential for punishment or an interaction with a friend. We should have performed suspiciousness checks to improve the quality of the data. In addition, our study on the effects of psychopathic traits on behavior was not controlled for demographic factors that might co-vary with levels of psychopathic traits (e.g., addiction, socio-economic status and intelligence). To improve our understanding of selective fair behavior as a function of psychopathic traits, further studies on mediators or moderators including emotionality, cognitive abilities, and empathy will be required.

Despite these limitations, the mean offers in the DG and the UG for individuals with higher- and lower tendencies toward primary psychopathy were not dramatically different from the corresponding data in previous studies that used real money (Koenigs et al., 2010; Gillespie et al., 2013). The moral dysfunction associated with psychopathy has been discussed by various studies with scenarios of social interactions including moral dilemmas (e.g., Koenigs et al., 2012). Although this approach is not ideal for obtaining conclusive answers, the findings in the present study offer material for discussion and support the argument that subclinical psychopathy may promote adaptive behaviors strategically during reciprocal social interactions.

## Ethics Statement

This study was carried out in accordance with the Japanese Ethical Guidelines for Medical and Health Research Involving Human Subjects. Informed consent from subjects was deemed provided upon completion of the questionnaire. The protocol was approved by the ethics committee for psychological research at Hiroshima Shudo University.

## Author Contributions

TO designed the study, collected and analyzed the data, and wrote the paper. TO and HO reviewed the manuscript and approved it for the final submission.

## Conflict of Interest Statement

The authors declare that the research was conducted in the absence of any commercial or financial relationships that could be construed as a potential conflict of interest.
