# Supplementary material for: Selective Fair Behavior as a Function of Psychopathic Traits in a Subclinical Population
Source: Front Psychol. 2017 Sep 13;8:1604. doi: 10.3389/fpsyg.2017.01604 (PMC5601322; doi:10.3389/fpsyg.2017.01604)
Supplement: Supplementary file 1 [file Data_Sheet_1.pdf]

# Scenario of the Dictator Game with a Stranger

Imagine that 1,000 yen is to be divided between you and a partner.

In this case, your partner is an unknown student at your university, who will never interact with you again.

You can make an offer regarding how the money will be distributed between you and the stranger.

On the other hand, the partner has no right to reject your offer.

Both you and your partner will receive the amount of money based on your offer even if your partner didn't accept your offer.

In this interaction, you can make just one offer.

What is your offer?

Out of 1,000 yen,

You get ____________________ yen

The stranger gets ____________________ yen

Please wait for further directions.

## Scenario of the Dictator Game with a Friend

Imagine that 1,000 yen is to be divided between you and a partner.

In this case, your partner is a friend at your university.

You can make an offer regarding how the money will be distributed between you and your friend.

On the other hand, the partner has no right to reject your offer.

Both you and your partner will receive the amount of money based on your offer even if your partner didn't accept your offer.

In this interaction, you can make just one offer.

What is your offer?

Out of 1,000 yen,

You get ____________________ yen

Your friend gets ____________________ yen

Please wait for further directions.

## Scenario of the Ultimatum Game with a Stranger

Imagine that 1,000 yen is to be divided between you and a partner.

In this case, your partner is an unknown student at your university, who will never interact with you again.

You can make an offer regarding how the money will be distributed between you and the stranger.

On the other hand, the partner has the right to reject your offer.

Neither you nor your partner will receive any money if your partner rejects your offer.

Instead, both of you will receive an amount of money based on your offer only if your partner accepts your offer.

In this interaction, you can make just one offer.

What is your offer?

Out of 1,000 yen,

You get ____________________ yen

The stranger gets ____________________ yen

Please wait for further directions.

## Scenario of the Ultimatum Game with a Friend

Imagine that 1,000 yen is to be divided between you and a partner.

In this case, your partner is a friend at your university.

You can make an offer regarding how the money will be distributed between you and your friend.

On the other hand, the partner has the right to reject your offer.

Neither you nor your partner will receive any money if your partner rejects your offer.

Instead, both of you will receive an amount of money based on your offer only if your partner accepts your offer.

In this interaction, you can make just one offer.

What is your offer?

Out of 1,000 yen,

You get ____________________ yen

Your friend gets ____________________ yen

**Please wait for further directions.**
